# A Narrative Review of Acanthamoeba Isolates in Malaysia: Challenges in Infection Management and Natural Therapeutic Advancements

**DOI:** 10.7759/cureus.72851

**Published:** 2024-11-01

**Authors:** Mohammad Wisman Abdul Hamid, Roslaini Bin Abd Majid, Victor Fiezal Knight Victor Ernest, Nik Noorul Shakira Mohamed Shakrin, Firdaus Mohamad Hamzah, Mainul Haque

**Affiliations:** 1 Medical Parasitology and Entomology, National Defence University of Malaysia, Kuala Lumpur, MYS; 2 Public Health and Military Medicine, National Defence University of Malaysia, Kuala Lumpur, MYS; 3 Medical Microbiology and Immunology, National Defence University of Malaysia, Kuala Lumpur, MYS; 4 Centre for Defence Foundation Studies, National Defence University of Malaysia, Kuala Lumpur, MYS; 5 Pharmacology and Therapeutics, National Defence University of Malaysia, Kuala Lumpur, MYS

**Keywords:** acanthamoeba, acanthamoeba keratitis (ak), amoeba-resistant bacteria (arbs), contact lens hygiene, cyst resilience, environmental contamination, granulomatous amoebic encephalitis (gae), nanotechnology, natural therapeutics, public health strategies

## Abstract

*Acanthamoeba*,* *a free-living amoeba (FLA) found in diverse ecosystems, poses significant health risks globally, particularly in Malaysia. It causes severe infectious diseases, e.g., *Acanthamoeba *keratitis (AK), primarily affecting individuals who wear contact lenses, along with granulomatous amoebic encephalitis (GAE), a rare but often life-threatening condition among immunocompromised individuals. AK has become increasingly prevalent in Malaysia and is linked to widespread environmental contamination and improper contact lens hygiene. Recent studies highlight *Acanthamoeba*’s capacity to serve as a “Trojan horse” for amoeba-resistant bacteria (ARBs), contributing to hospital-associated infections (HAIs). These symbiotic relationships and the resilience of *Acanthamoeba* cysts make treatment challenging. Current diagnostic methods in Malaysia rely on microscopy and culture, though molecular procedures like polymerase chain reaction (PCR) are employed for more precise detection. Treatment options remain limited due to the amoeba’s cyst resistance to conventional therapies. However, recent advancements in natural therapeutics, including using plant extracts such as betulinic acid from *Pericampylus glaucus* and chlorogenic acid from *Lonicera japonica*, have shown promising in vitro results.

Additionally, nanotechnology applications, mainly using gold and silver nanoparticles to enhance drug efficacy, are emerging as potential solutions. Further, in vivo studies and clinical trials must validate these findings. This review highlights the requirement for continuous research, public health strategies, and interdisciplinary collaboration to address the growing threat of *Acanthamoeba* infections in Malaysia while exploring the country’s rich biodiversity for innovative therapeutic solutions.

## Introduction and background

Globally, *Acanthamoeba* has surfaced as a considerable public health distress because of its capacity to cause serious human diseases. Although such infections are relatively rare, the consequences can be devastating, particularly with conditions like *Acanthamoeba* keratitis (AK), which primarily affects contact lens wearers, and granulomatous amoebic encephalitis (GAE), a rare but frequently life-threatening brain infection in individuals with compromised immune systems​ [1-3]. The growing use of contact lenses around the globe, coupled with the pathogen’s resilience in various environments, has heightened the relevance of *Acanthamoeba* as a critical pathogen. The amoebae’s ability to thrive in diverse habitats, ranging from natural water sources to man-constructed environs, e.g., air conditioning systems and pools, highlights their potential as a persistent and widespread threat to human health. Although countries with robust public health infrastructures continue to face challenges in managing *Acanthamoeba*-related infections, the situation in Malaysia is particularly concerning due to a combination of environmental and infrastructural factors [4-11].

In Malaysia, the presence of *Acanthamoeba* has been extensively documented across various environmental sources, showing a substantial prevalence in water supplies, soil, and indoor contexts. Studies have shown that *Acanthamoeba* contamination is alarmingly high in those lakes and rivers and attracts tourists and holidaymakers, with some locations exhibiting a 100% positivity rate [6]. Artificial environments, including swimming pools and domestic tap water, have shown notable contamination rates, indicating that these amoebae can endure even with routine maintenance and water treatment procedures [4,7]. This broad environmental distribution increases the likelihood of human exposure, particularly for contact lens users and individuals with compromised immune systems, placing Malaysia at greater risk than many other regions.

The hospital setting presents an additional layer of concern [9]. *Acanthamoeba* has been isolated from various water systems within hospitals, including those servicing surgical units, intensive care units (ICUs), operating rooms, and water storage tanks, with a contamination rate of 53.5% [12]. Further studies have shown even higher contamination rates, with 90% of samples from water and biofilms in sterilization service units, central sterilization service units, and endoscopy/bronchoscopy units testing positive for *Acanthamoeba* [13,14]. Additionally, “dry” or “moist” biofilms found on floors and sink outlets had a 76.7% positivity rate [15], and dust samples from the hospital’s ventilation and air conditioning systems showed a 77.8% contamination rate [11]. These findings emphasize the continuing threat of infection by highlighting the widespread presence of *Acanthamoeba* in both public and hospital settings.

**Figure 1:**
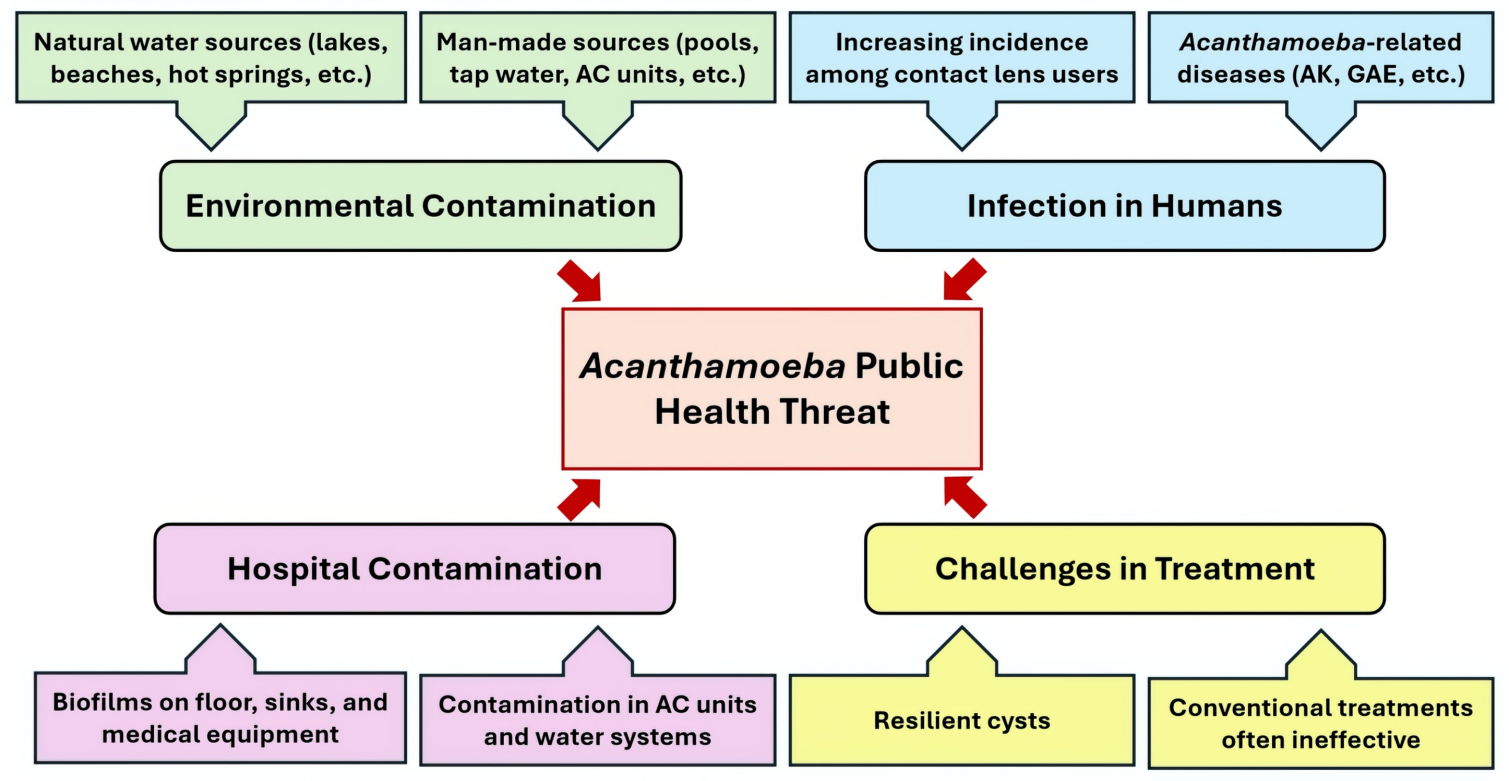
The public health risks associated with Acanthamoeba Image Credit: Mohammad Wisman Abdul Hamid

Clinically, *Acanthamoeba*-related diseases typically present as AK, a distressing and potentially vision-threatening condition that mainly affects individuals who wear contact lenses [16]. This condition, frequently associated with inadequate lens hygiene, can result in serious complications, including permanent vision loss, if not swiftly diagnosed and managed [2,17]. Managing AK is difficult, mainly because of the challenging nature of *Acanthamoeba* cysts, which are resistant to a wide range of standard treatments [18-20]. This highlights the critical need for early detection and intervention to prevent disease progression. In Malaysia, the frequency of AK has been rising, in parallel with the growing popularity of contact lens use, especially among younger populations [21]. This trend emphasizes the importance of public health education on proper lens hygiene and the risks associated with *Acanthamoeba* exposure. Although GAE and other rare *Acanthamoeba*-related diseases have not yet been reported in Malaysia, they remain a serious concern due to their high mortality rates and potential impact on immunocompromised individuals. Figure 1 illustrates the public health risks posed by *Acanthamoeba* in Malaysia.

Problem statement of the review

Despite global recognition of *Acanthamoeba* infections as a severe health concern, Malaysia has a substantial gap in clinical awareness and environmental control [22-25]. Limited public knowledge and the challenges in diagnosing and treating these infections, mainly due to the resilience of *Acanthamoeba* cysts, pose significant risks to public health. With the increasing prevalence of *Acanthamoeba* in both natural and artificial environments, there is an urgent need for focused research and more potent public health strategies to reduce infection rates and improve management. Immediate action is required to address these gaps and develop effective prevention, early detection, and treatment solutions.

Objective of the study

This review seeks to present a comprehensive summary of *Acanthamoeba* infections within Malaysia, focusing on the key challenges in diagnosing, treating, and controlling the spread of this protozoa. Specifically, it seeks to explore current diagnostic limitations, the need for more effective treatment strategies to combat cyst resilience, and the role of environmental control in reducing exposure risks. Additionally, the review will highlight recent advancements in natural therapeutics and nanotechnology to identify innovative solutions for both clinical management and public health interventions. The review aims to promote further research and practical applications to reduce the public health impact of *Acanthamoeba* infections in Malaysia by addressing these critical areas.

## Review

Materials and methods

**Figure 2:**
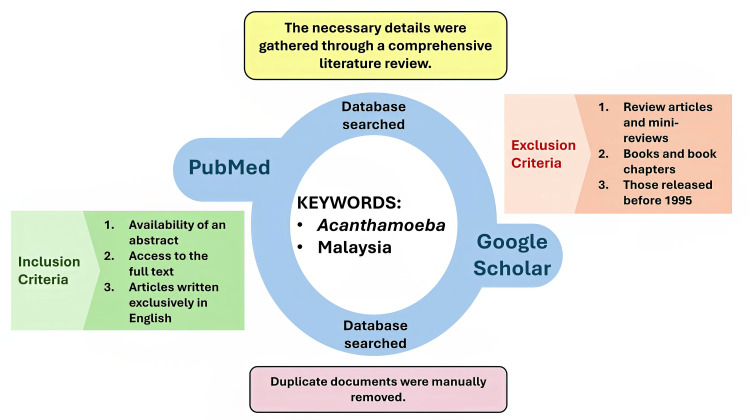
A chart illustrating the methodology used in this paper Image Credit: Mohammad Wisman Abdul Hamid

A thorough search was conducted through PubMed and Google Scholar to find studies on the environmental and clinical isolation of *Acanthamoeba* species in Malaysia up to 1995 (Figure 2). The search terms “Acanthamoeba” AND “Malaysia” were used to gather relevant studies. Critical information was extracted from the selected articles, such as the year of the study, location, sample types, identification methods, isolated *Acanthamoeba *strains, clinical symptoms, and references. Each paper’s relevance was carefully assessed before being included in the study. After an independent review of the selected literature, a follow-up discussion was held to resolve questions, correct errors, and address potential biases in the findings.

Review of literature

An Overview of Acanthamoeba Biology

**Figure 3:**
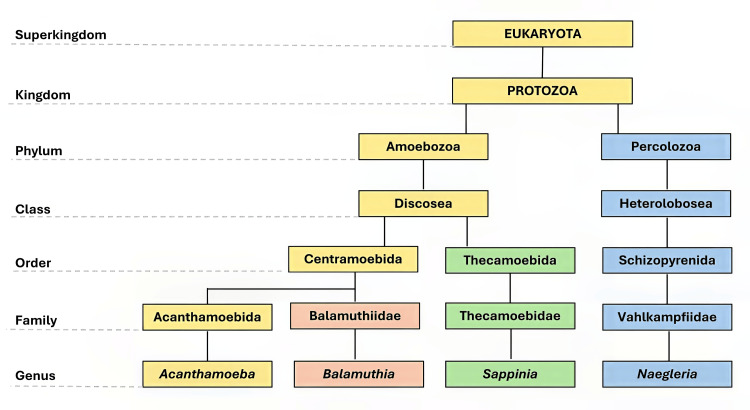
Taxonomic classification of Acanthamoeba, along with Balamuthia, Sappinia, and Naegleria Image Credit: Mohammad Wisman Abdul Hamid

*Acanthamoeba* was first identified in the 1930s [26], but it wasn’t until the 1970s that it was recognized as a pathogen affecting humans, particularly in cases of keratitis linked to contact lens use [27-29]. The word “acanth” (from the Greek “acanth” indicating “spikes”) was incorporated into the word “amoeba” to denote the presence of spike-like projections on its surface, which are today called acanthopodia [1,29]. Acanthamoeba belongs to the kingdom Protozoa, phylum Amoebozoa, class Discosea, order Centramoebida, and family *Acanthamoebidae* (Figure 3) [30]. *Acanthamoeba* spp. are classified into species based on morphological features and molecular characteristics. 

*Acanthamoeba*’s morphological categorization primarily relies on their cysts’ features, classified as group I, group II, and group III [31]. Group I is characterized by large cysts, generally having an average diameter of 18 µm or more. The ectocyst in this group is usually rounded and either smooth or slightly wrinkled, while the endocyst often has a stellate shape. The ectocyst and endocyst are noticeably spaced apart, with the endocyst connecting to the ectocyst at the ends of its rays or arms [32-34]. Species within this group include *Acanthamoeba astronyxis*, *A. byersi*, *A. comandoni*, *A. tubiashi*, and *A. echinulate* [35]. Group II comprises comparatively smaller cysts, typically with an average diameter of less than 18 µm. The ectocyst can be thick or thin in this group and often appears wrinkled. At the same time, the endocyst can have various shapes, including stellate, polygonal, triangular, round, or oval, typically lacking well-defined arms or rays. The spacing between the ectocyst and endocyst differs, ranging from being closely positioned to widely separated [32-34]. This group is the most frequently isolated and contains several pathogenic *Acanthamoeba* species, including *A. castellanii, A. polyphaga*,* A. hatchetti*,* A. rhysodes*, *A. mauritaniensis*,* A. griffini*, and *A. micheli* [35]. Group III consists of small cysts, typically averaging less than 18 µm in diameter. The ectocyst in this group is thin and can either be smooth or slightly rippled. The endocyst is generally round, often featuring three to five faintly defined corners, making the outer wall challenging to discern [32-34]. Species in this group include *A. lugdunensis*, *A. quina*, *A. triangularis*, *A. divionensis*,* A. stevensoni,* and *A. gigantea* [35].

In recent years, molecular classification has become increasingly important for categorizing *Acanthamoeba* into genotypes using 18S rRNA gene sequence data [34]. Twenty-three genotypes (T1-T23) have been recognized [36]. Research indicates that these genotypes are closely linked to pathogenicity, with each genotype associated with unique disease presentations and pathological characteristics [34,37]. The T4 genotype is the most frequently detected in the environment and is associated with the most serious clinical cases of *Acanthamoeba* infections, with the T3 genotype following closely behind [35,38]. AK is associated with genotypes T2, T4, T5, T6, T10, T11, T12, and T15 [38-40], whereas genotypes T1, T2, T4, T5, T10, T11, and T12 are linked to GAE [39,41]. Genotypes T2, T4, T5, T16, and T18 have also been associated with pulmonary acanthamoebiasis [41,42]. This molecular approach has enhanced the clarity and precision of *Acanthamoeba* classification, effectively addressing the limitations of traditional morphological methods. Currently, morphological classification and molecular characterization are often used to provide a comprehensive understanding of *Acanthamoeba* species [43].

**Figure 4:**
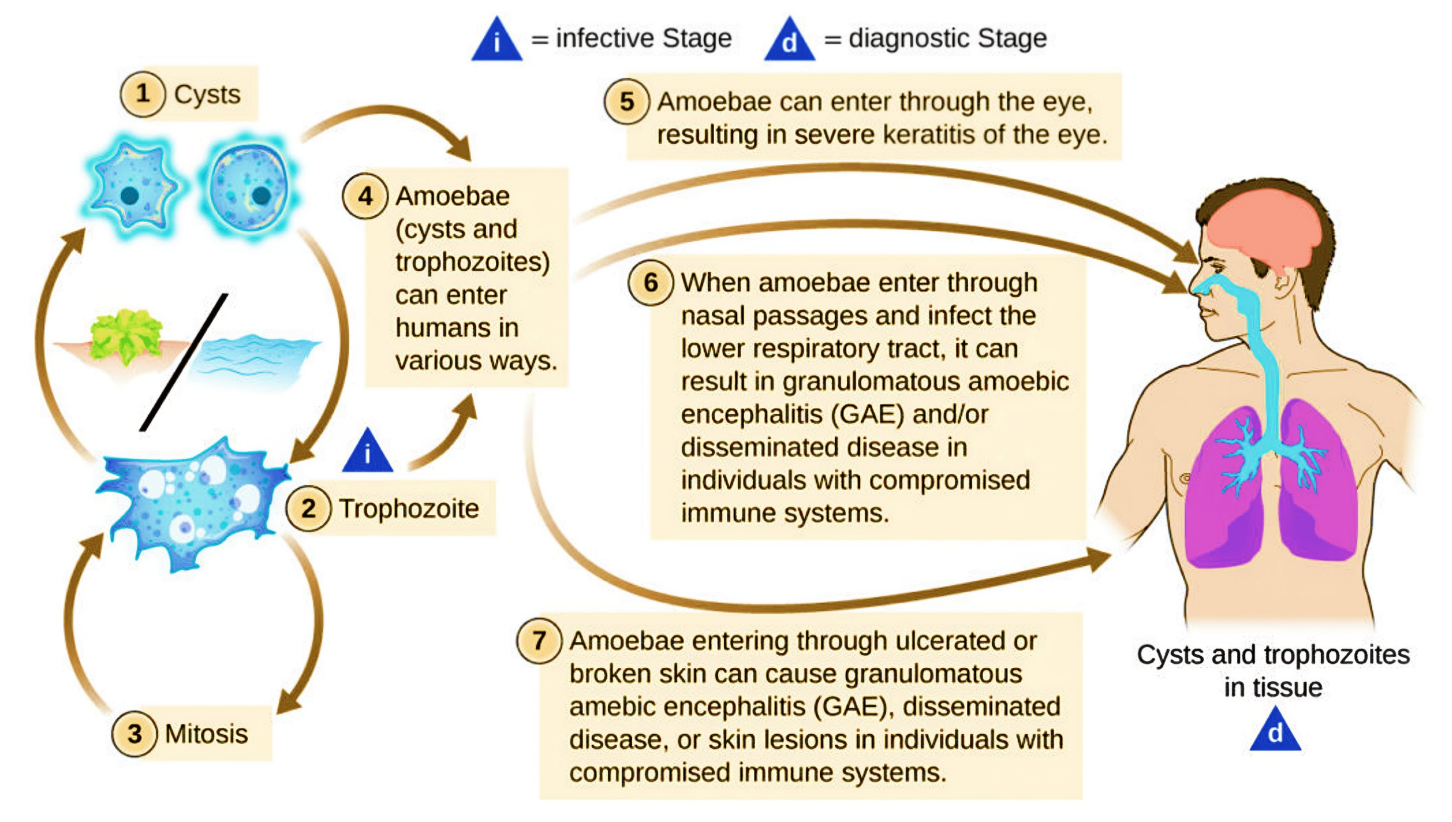
Lifecycle of Acanthamoeba and its transmission routes to humans Notes: The concept of this figure was acquired from work by the Centers for Disease Control and Prevention (CDC) (https://courses.lumenlearning.com/suny-microbiology/chapter/protozoan-and-helminthic-infections-of-the-skin-and-eyes/). This figure of CDC is under open license, free to modify, share, and use commercially. One of our team members adjusted the image. Image Credit: Mohammad Wisman Abdul Hamid

*Acanthamoeba*’s life cycle (Figure 4) consists of two primary stages: the trophozoite and the cyst [1,26]. The trophozoite, ranging in size between 25 and 40 µm, generally displays a long, oval, or irregular form with unclear cytoplasmic boundaries [21,44,45]. It is characterized by pseudopods crucial for movement, feeding, and adhesion, mainly to contact lenses, which increases the risk of corneal infections [35,46]. While the trophozoite is generally uninucleate, it can become multinucleated under specific conditions, such as in agitated aquatic environments, facilitating colonization and reproduction [47]. Internally, the trophozoite contains organelles such as mitochondria, ribosomes, and vacuoles. Prominent among these vacuoles are the contractile vacuoles, which regulate osmotic balance, and digestive vacuoles, which decompose ingested particles. These features contribute to the amoeba’s adaptability and survival across various environments. *Acanthamoeba* trophozoites primarily feed on bacteria, algae, yeast, and small organic particles through phagocytosis or pinocytosis, creating multiple food vacuoles within their cytoplasm [1,35].

Under adverse conditions like nutrient depletion or extreme temperatures, *Acanthamoeba *transforms into a cyst, a dormant and highly resilient form capable of surviving in harsh environments for extended periods [1,26,48]. These cysts, typically round and measuring 13-20 µm in diameter, are protected by a double-layered cell wall, with the outer ectocyst composed of laminar fibrous material and the inner endocyst made of fine fibers, primarily cellulose. The tightly packed microfibrils in the cyst wall provide exceptional tensile strength and durability, making the cysts resistant to degradation and various environmental stresses [1,32,34,49]. The transition from trophozoite to cyst is triggered by unfavorable conditions such as nutrient deprivation, desiccation, and extreme temperatures. Remarkably, *Acanthamoeba* cysts can remain viable for decades, with studies showing survival in dry environments for up to 21 years and in water at 4°C for over 24 years. These cysts are also highly resistant to fungicides, chlorinating agents, and antibiotics, complicating the treatment of infections. Long-term studies have confirmed the resilience and virulence of *Acanthamoeba *cysts, with some strains maintaining high levels of pathogenicity even after 24 years [35,50,51].

*Acanthamoeba* utilizes various mechanisms to colonize, invade, and inflict considerable damage on host tissues, especially in individuals with weakened immune systems. The infection process starts with the attachment to host epithelial cells, aided by acanthopods and surface adhesins like mannose-binding proteins (MBPs) and laminin-binding proteins (LBPs). Pathogenic strains show increased acanthopods and greater expression of MBPs, enhancing their ability to adhere [52-54]. Following adhesion, *Acanthamoeba* triggers intracellular signaling cascades that lead to phagocytosis of host cells and secretion of proteases, which degrade the extracellular matrix and facilitate deeper tissue invasion [35]. Additionally, *Acanthamoeba* can induce apoptosis in host cells through various signaling pathways, further contributing to tissue damage in conditions like AK and GAE [35,52]. The secretion of proteolytic enzymes also contributes to the lysis of host cells, intensifying the infection’s severity [55-58].

Furthermore, *Acanthamoeba *has evolved immune evasion mechanisms that enable it to survive within immune cells and resist destruction, allowing it to persist and spread within the host, which complicates the treatment and management of infections [26,35]. The study also demonstrated that *Acanthamoeba *is frequently linked to environmental biofilms, which have been shown to promote amoeba infections. The biofilm acts as a protective environment for *Acanthamoeba*, aiding in immune evasion, enhancing its invasiveness, and possibly even supplying it with nutrients [26,38,59,60].

An essential aspect of *Acanthamoeba*’s biology is its symbiotic interaction with amoeba-resistant bacteria (ARBs), which reside within the amoeba as endosymbionts. *Acanthamoeba* acts as a “Trojan horse,” serving as a reservoir and a vehicle for pathogenic bacteria, shielding them from environmental stresses and facilitating their proliferation [61]. *Acanthamoeba*’s role as a host for pathogenic microorganisms was first identified in the 1970s [62]. 

*Acanthamoeba* can harbor various environmental bacteria, including *Mycobacterium *avium subsp. *Paratuberculosis* [39,63-66]. These ARBs can survive within *Acanthamoeba* and be released in expelled food vacuoles (EFVs), where they remain protected until they encounter a suitable environment for proliferation [66,67]. Within *Acanthamoeba* cells, these pathogens can endure harsh environmental conditions, such as those experienced during water treatment or exposure to hospital disinfectants. Notably, this group includes members of the ESKAPE pathogens (*Enterococcus faecium, Staphylococcus aureus, Klebsiella pneumoniae, Acinetobacter baumannii, Pseudomonas aeruginosa, and Enterobacter* species), which are well-known for causing hospital-associated infections (HAIs) and exhibiting significant antibiotic resistance [68,69]. In Malaysia, a single study has investigated ARBs, finding that out of 36 *Acanthamoeba *isolates from dust samples taken from air-conditioning systems in operating theaters and wards at Universiti Kebangsaan Malaysia Medical Centre (UKMMC), *Mycobacterium* spp.* *(82.76%), *Legionella* spp.* *(65.52%), and *Pseudomonas* spp.* *(62.07%) were identified as ARBs [70].

Acanthamoeba-Related Diseases

*Acanthamoeba* is an opportunistic free-living amoeba (FLA) that can lead to various human infections, such as AK, GAE, disseminated infections, and skin lesions, particularly in individuals with weakened immune systems. The recognition of the pathogenic potential of *Acanthamoeba* dates to the late 1950s, with critical observations made in vitro using monkey kidney cells and experimental inoculation in animal models, including monkeys and mice [45,71,72]. The first human case of GAE, a rare but often fatal brain infection, was reported in 1972 [28,73]. Cases of AK followed this in 1974 [27,43]. Initially considered rare pathogens, *Acanthamoeba *spp. are now recognized as causative agents of various infections, including skin lesions, sinusitis, AK, and GAE. These infections predominantly affect individuals with compromised immune systems, although AK is frequently reported in otherwise healthy individuals, particularly contact lens wearers [17,26,74,75].

AK is the most frequently occurring illness caused by *Acanthamoeba*, often linked to corneal trauma, contaminated water exposure, and contact lens use [18,76]. Contact lenses may create minor abrasions on the cornea, heightening the risk of infection as *Acanthamoeba* trophozoites attach to the surface of the lenses [77]. Early silicone hydrogel lenses were prone to trophozoite attachment, but second-generation lenses have reduced this issue [78,79]. However, some lens materials may still promote cyst formation, complicating disinfection [80]. Infection typically starts in the corneal epithelium and can progress to the stroma, usually affecting one eye [26,48,81]. Symptoms include redness, tearing, blurred vision, and severe pain. Symptoms include redness, excessive tearing, blurred vision, and intense pain. If left untreated, AK can result in severe complications such as corneal perforation [2,17]. Inadequate contact lens hygiene is a significant risk factor for developing AK. Using tap water to rinse lenses, “topping off” old disinfectant, and failing to clean storage cases properly can all lead to contamination [4,82-84]. Risky behaviors such as rubbing eyes while wearing or sleeping with lenses increase the chances of developing AK [35,85].

GAE is an uncommon but usually lethal infection affecting the central nervous system, with a mortality rate exceeding 90% despite its low global incidence [3,42]. It mainly impacts individuals with weakened immune systems, such as those with HIV/AIDS, organ transplant recipients, or patients receiving cancer treatments. However, it can also occur in otherwise healthy individuals [73,86]. GAE typically manifests as subacute or chronic granulomatous encephalitis, characterized by symptoms such as neck stiffness, headache, fever, and worsening neurological problems, including altered mental status, seizures, confusion, hallucinations, cranial nerve palsies, ataxia, paralysis, and personality changes [35,87-89]. The disease advances quickly, typically leading to death within one to two months due to elevated intracranial pressure [90]. Although the exact pathogenesis of GAE remains unclear, it is thought that *Acanthamoeba* enters the body through the respiratory system or skin lesions, then spreads to the brain through the bloodstream [3,90]. The olfactory epithelium may also serve as a pathway for the amoeba to reach the central nervous system [3]. Due to its rarity and often non-specific symptoms, GAE is frequently underdiagnosed, making early clinical suspicion and specialized laboratory expertise crucial for timely diagnosis and treatment [91]. A significant challenge in treating GAE is *Acanthamoeba*’s ability to encyst during infection, as these cysts are highly resistant to most antimicrobial agents [3,73,87,92].

*Acanthamoeba* can also cause several other rare conditions, including cutaneous acanthamoebiasis (CA), *Acanthamoeba *rhinosinusitis (AR), and *Acanthamoeba *pneumonia (AP). The earliest recorded case of CA occurred in 1986 in a patient with AIDS [93,94]. CA typically presents as spots or single or multiple nodules that can enlarge over time and eventually ulcerate. These skin lesions usually develop on the face, torso, and limbs, making diagnosis challenging due to the condition’s varied clinical presentation [33,94]. AR occurs exclusively in immunocompromised individuals, especially those with HIV. Symptoms of AR include nasal blockage, crusting, and nosebleeds.

Additionally, necrosis of the bone and cartilage within the nasal cavity may occur, surrounded by swollen, reddened mucosa, potentially leading to osteomyelitis, particularly in the bones of the hard palate [95]. AP primarily affects immunocompromised individuals, presenting with symptoms such as weight loss, reduced respiratory function, and pulmonary edema, as detected in radiological exams [35]. In addition to these conditions, *Acanthamoeba *can infect other organs, including the liver, adrenal glands, kidneys, lymph nodes, breasts, heart, spleen, testicles, thyroid, and urethra. However, such infections are sporadic [96].

Laboratory Techniques for the Detection of Acanthamoeba

Culture methods remain foundational for detecting *Acanthamoeba*, though their sensitivity varies. The most widely used culture technique globally involves non-nutrient agar (NNA) overlaid with *Escherichia coli *to encourage the growth of *Acanthamoeba*, as *E. coli* provides nourishment for the trophozoites. Trophozoites are generally observed at the ends of the visible migration paths [97-99]. However, the sensitivity of this method can range significantly, from 33.3% to 66.7%, depending on sample handling, culture conditions, and technique used [99-101]. Storing samples in a liquid medium like PYG (peptone, yeast extract, and glucose) at 4°C can effectively preserve them for extended periods, potentially ranging from one to four years [45,102].

Microscopy is essential for identifying *Acanthamoeba*, particularly in resource-limited settings where advanced molecular techniques are unavailable. Light microscopy is commonly used to initially detect *Acanthamoeba* cysts and trophozoites in clinical and environmental samples. Staining techniques are frequently utilized to enhance the visualization of trophozoites and cysts. A study found that the iodine stain, which achieved the highest score of 92%, was the most balanced option overall, excelling in handling, cost, and time efficiency [103]. The Gimenez stain, with a score of 76%, provides excellent visual results but is less practical due to higher costs and more complex handling. Methylene blue, scoring 72%, offers moderate performance, making it a reasonable choice when higher-performing stains are unavailable. Giemsa stain, scoring the lowest at 44%, is the least preferred due to poor staining results despite being more accessible and cost-effective.

Other stains used for detecting trophozoites and cysts include modified trichrome, calcofluor white (CFW), hematoxylin and eosin (H&E), periodic acid-Schiff (PAS), and Gömöri methenamine silver. On the other hand, stains like Gram, acridine orange, and Giemsa are considered some of the least effective [43,103-105]. When observed under a microscope with staining, cysts typically exhibit double-walled structures, with the endocyst (inner wall) appearing as a distinct layer separate from the ectocyst (outer wall). The endocyst can assume different shapes, such as stellate, oval, round, or polygonal, while the ectocyst is seen against a background of varying stain intensity [103].

Molecular methods, especially polymerase chain reaction (PCR), have transformed the detection of *Acanthamoeba*, providing greater sensitivity and specificity compared to conventional techniques. PCR is the most commonly used molecular method, explicitly targeting the 18S rRNA, an essential part of the 40S small ribosomal subunit [21,106,107]. The sensitivity of PCR for detecting Acanthamoeba varies, ranging from 73.3% in single PCR assays to as high as 93.3-100% in combined PCR assays. These combined assays also achieve a specificity of 99.3-100%, establishing PCR as the gold standard for *Acanthamoeba *detection [99,108,109]. Real-time or quantitative PCR (qPCR) has become increasingly popular in recent years because it allows for the real-time monitoring of DNA amplification by measuring fluorescence levels [110]. This method dramatically reduces processing time, with results available in as little as 3.5 hours [110-112]. One of the critical advantages of qPCR is the elimination of post-amplification processing, which not only accelerates the overall analysis but also minimizes the risk of contamination from amplified products.

Additionally, qPCR provides a quantitative assessment of pathogen load, offering valuable insights into the extent of infection [43,113]. Multiplex PCR allows for the simultaneous amplification of several DNA sequences, enabling the identification of multiple organisms within a single test [114]. Additional advanced methods used to diagnose Acanthamoeba infections include loop-mediated isothermal amplification (LAMP), nested PCR, and nanoparticle-assisted PCR (nanoPCR) [115-117]. Furthermore, Next-Generation Sequencing (NGS) is an evolving technology that provides comprehensive genetic analysis of Acanthamoeba populations within a sample. A study found that an NGS-based assay successfully identified *Acanthamoeba*-specific sequences with 100% specificity and 88% sensitivity [118].

Management of Acanthamoeba Infections

Treating AK is challenging due to the organism’s resilience, particularly its cystic form, which is resistant to many treatments. The primary treatment consists of using topical antiseptic agents such as 0.02-0.08% polyhexamethylene biguanide (PHMB) or 0.02-0.06% chlorhexidine, in combination with a topical diamidine like 0.1% propamidine isethionate [119-121]. These agents increase membrane permeability, killing cysts and trophozoites [122,123]. In severe cases, neomycin is combined with biguanides to prevent bacterial infections. Treatment typically lasts several months, starting with frequent applications that taper off as the infection stabilizes [2,124]. Antifungal agents like voriconazole or posaconazole may be used if the infection persists, though concerns about drug toxicity and cost remain [16,125]. Some cases may require corneal transplantation, but surgery is usually delayed until the infection is under control to prevent recurrence [16].

Treating GAE is even more complex due to its high mortality rate exceeding 90% [96]. No single treatment is considered definitive, and therapy typically involves a combination of medications such as sulfadiazine, pentamidine, fluconazole, flucytosine, and miltefosine, as the CDC advises. These drugs target both the amoeba’s trophozoite and cyst forms. However, the medications can have severe side effects, such as nephrotoxicity and bone marrow suppression. Newer drugs like nitroxoline have shown promise, but access to these treatments remains challenging. In some cases, surgery may be needed to reduce the amoebic load, but due to GAE’s rarity, most treatment protocols are based on individual case reports [73,96].

Preventing *Acanthamoeba *infections through disinfection is also tricky due to the cysts’ resistance. Studies have shown that quaternary ammonium compounds (QACs) and octenidine dihydrochloride (OCT) are most effective, killing both trophozoites and cysts within one minute. Alcohol-based disinfectants are less effective, only acting on trophozoites. Agents like peracetic acid and potassium peroxymonosulfate need longer exposure times to kill cysts. These findings suggest that QAC- and OCT-based products offer better solutions for disinfection, particularly in healthcare settings [126].

Discussion

Acanthamoeba Distribution in Malaysia

The examination of *Acanthamoeba*-positive samples in Malaysia indicates a troubling presence of this FLA in diverse environmental sources, posing notable public health concerns (Table 1). An analysis of 42 domestic tap water samples from Kuala Lumpur revealed a positivity rate of 2.4% (n = 1) [4], highlighting the presence of *Acanthamoeba* spp. in treated water supplies, which presents a potential risk to the public [4]. An even more alarming finding is the positivity rate of 46.19% (n = 388) out of 840 swimming pool water samples tested in Kuala Lumpur [127], which highlights the resilience of *Acanthamoeba* in recreational water bodies, even under regular maintenance and water treatment conditions.

**Table 1:** Acanthamoeba distribution in the Malaysian environment

Sample type	Location/state	Total samples	Positive samples (%)	Laboratory investigations	Identified strains	References
Water samples from domestic tap water	Kuala Lumpur	42	1 (2.4%)	Culture and microscopy	*Acanthamoeba *spp.	[4]
Dust from air conditioning units	International Medical University (IMU), Kuala Lumpur	87	20 (23%)	Culture, microscopy, and PCR	A. castellanii, A. culbertsoni, A. griffini, A. lenticulate, A. polyphaga	[5]
Water samples from 15 recreational rivers	Kuala Lumpur and Selangor	15	15 (100%)	Culture and microscopy	A. lenticulata	[6]
Swabs from rocks and stones	Kuala Lumpur	15	11 (73.33%)	Culture and microscopy	A. graffini, A. polyphaga, A. A. lenticulata, A. jacobsi	
Wet soils	Kuala Lumpur	15	15 (100%)	Culture and microscopy	A. castellanii	
A swab from outdoor wall surfaces	Medical Faculty of Universiti Malaya (UM), Kuala Lumpur	20	20 (100%)	Culture and microscopy	A. castellanii, A. polyphaga	
A swab from indoor wall surfaces		20	20 (100%)	Culture and microscopy	A. castellanii, A. jacobsi	
Water samples from drinking water treatment plant	Sarawak	61	55 (90.2%)	Culture, microscopy, and PCR	A. castellanii, A. griffini	[25]
Water samples from tap water	All states in Peninsular Malaysia	181	45 (24.9%)	Culture and PCR	*Acanthamoeba *spp.	[128]
Water samples from the recreational area		57	40 (70.2%)	Culture and microscopy	*Acanthamoeba *spp.	
Water samples from the water dispenser		3	2 (66.7%)	Culture and microscopy	Acanthamoeba spp.	
Water samples from rice paddy field		4	4 (100%)	Culture and microscopy	*Acanthamoeba* spp.	
Water samples from Sungai Klah hot spring	Perak	10	7 (70%)	Culture, microscopy, and molecular	T3-T5	[129]
Water samples from Hulu Tamu hot spring	Selangor	10	10 (100%)	Culture, microscopy, and molecular	T4 and T15	
Water samples from the Selayang hot spring	Selangor	10	5 (50%)	Culture, microscopy, and molecular	T4	
Water samples from Bentong hot spring	Pahang	10	7 (70%)	Culture, microscopy, and molecular	T4, T11, T15, and T17	
Water samples from the Gadek hot spring	Melaka	10	9 (90%)	Culture, microscopy, and molecular	T4 and T15	
Water samples from Pantai Morib beach	Selangor	50	45 (90%)	Culture, microscopy, and molecular	T4, T11, and T18	[130]
Water samples from Pantai Teluk Kemang beach	Negeri Sembilan	50	40 (80%)	Culture, microscopy, and molecular	T4 and T5	
Water samples from Pantai Teluk Batik beach	Perak	50	30 (60%)	Culture, microscopy, and molecular	T4 and T20	
Water samples from Pantai Tanjung Bidara beach	Melaka	50	30 (60%)	Culture, microscopy, and molecular	T4	
Water samples from Pantai Teluk Cempedak beach	Pahang	50	35 (70%)	Culture, microscopy, and molecular	T4, T11, T15, and T18	
Soil samples from Payeh Maga highland forest	Sarawak	8	8 (100%)	Culture, microscopy, and molecular	*Acanthamoeba* sp. boxer dog (T1), *A. polyphaga* strain	[131]
Water samples from the swimming pool	Kuala Lumpur	840	388 (46.19%)	Culture and microscopy	*Acanthamoeba *spp.	[127]
Water samples from Biru Lake	Selangor	10	10 (100%)	Culture, microscopy, and molecular	T4, T17, and T18	[132]
Water samples from Titiwangsa Lake	Kuala Lumpur	10	8 (80%)	Culture, microscopy, and molecular	T4 and T9	
Water samples from Shah Alam Lake	Selangor	10	8 (80%)	Culture, microscopy, and molecular	T4, T5, T11, T17, and T18	

Dust samples from air conditioning units at the International Medical University (IMU) in Kuala Lumpur showed a positivity rate of 23% (n = 20) out of 87, with multiple strains, including *A. culbertsoni* and *A. griffini*, identified through culture, microscopy, and PCR methods [5]. This highlights the potential for airborne transmission in indoor settings [5]. In a study conducted at the Medical Faculty of Universiti Malaya (UM), swabs from both outdoor and indoor wall surfaces in Kuala Lumpur demonstrated a contamination rate of 100% (n = 20), with identified strains including *A. castellanii*, *A. polyphaga*, and *A. jacobsi *[6]. These findings underscore the persistence of *Acanthamoeba* in controlled indoor environments, posing a risk in healthcare settings where sterility is crucial.

Water samples from a drinking water treatment plant in Sarawak exhibited a striking positivity rate of 90.2% (n = 55) out of 61, with *A. castellanii *and *A. griffini* strains identified [25]. This finding is particularly concerning as it points to the potential for widespread dissemination of *Acanthamoeba* through public water supplies [25]. Likewise, water samples from 15 recreational rivers in Selangor and Kuala Lumpur exhibited a positivity rate of 100% (n = 15), with *A. lenticulata* being the most commonly detected strain [6]. Additionally, swabs from stones and wet soil from these recreational rivers yielded positive results in 73.33% (n = 11) and 100% (15) out of 15 studied samples, respectively, with multiple strains, including *A. castellanii*, *A. polyphaga*, and *A. griffini*, being identified [6] with *A. lenticulata* being the most commonly detected strain [6]. 

A more extensive survey encompassing all states in Peninsular Malaysia found that 24.9% (n = 45) out of 181 tap water samples were positive for *Acanthamoeba* spp., and 70.2% (n = 40) out of 57 water samples from recreational sites were contaminated [128]. Interestingly, all water samples from rice paddy fields tested positive, highlighting the ubiquity of *Acanthamoeba* in agricultural environments [128]. Additionally, a study examining hot springs in Malaysia revealed high contamination rates across various locations, with positivity rates ranging from 50% (n = 5) out of 10 water samples [129]. The detected strains encompassed multiple genotypes, including T3, T4, T5, T11, T15, and T17, emphasizing the considerable genetic diversity of *Acanthamoeba* in these environments [129].

Recent research on water samples from beach locations, including Pantai Morib Beach, Pantai Teluk Kemang Beach, and Pantai Tanjung Bidara Beach, revealed contamination rates ranging from 60% (n = 30) to 90% (n = 45) out of 50 water samples, with genotypes T4, T5, T11, T18, and T20 identified [130]. These results indicate a potential public health risk for visitors to these recreational areas [130]. Soil samples from the Payeh Maga highland forest in Sarawak also demonstrated a positivity rate of 100% (n = 8) examined samples, with *A. polyphaga* and rare strains such as *Acanthamoeba sp*. boxer dog (T1) being detected, further supporting the extensive distribution of Acanthamoeba across various ecological environments [11,131,132].

The Clinical Significance and Health Impacts of Acanthamoeba in Malaysia

AK is Malaysia’s most reported *Acanthamoeba* infection, with the first case documented in 1995 involving a female contact lens wearer [133]. This infection is characterized by severe eye pain, redness, blurred vision, and, in advanced cases, blindness [17,43,48]. AK is mainly linked to improper contact lens hygiene, including rinsing lenses with tap water or insufficiently cleaning storage cases [19].

The analysis of *Acanthamoeba* infections in Malaysia from various hospitals between 2002 and 2015 reveals essential trends in the prevalence and associated risk factors (Table 2). In another study conducted from June to December 2002, 9.1% (n = 4) of 44 corneal scraping samples tested positive for *Acanthamoeba*, predominantly affecting female patients aged 23-46, with most cases linked to contact lens use [134]. The following year, the positivity rate increased to 14.87% (n = 11) out of 74 investigated samples. Furthermore, out of 11 positive cases, 10 were female, again primarily associated with contact lens usage [135]. Between 2010 and 2012, the positivity rate remained at 15.6% (n = 10) out of 64 samples, indicating a consistent risk among contact lens users [136]. However, the period from 2013 to 2015 saw a significant rise in *Acanthamoeba *detection, with a positivity rate of 22.6% (n = 14) out of 62 samples, with most cases occurring in individuals aged 19-35 years [137]. During these times, the primary risk factor was contact lens usage, highlighting the urgent need for better hygiene practices and increased public awareness to prevent these infections.

**Table 2:** Retrospective study findings of AK in Malaysia

Year	Locations	Samples	Positive samples (%)	Total samples	Total positive samples (%)	Age of positive cases (years old)	Sex of positive cases	Source of infection	Clinical samples	Laboratory investigations	Acanthamoeba group/strains	References
June-December 2002	Hospital Universiti Kebangsaan Malaysia	3	1 (33.3%)	44	4 (9.1%)	23-46	Male = 1, female = 3	Contact lens (3 patients); eye trauma (1 patient)	Corneal scrapings	Culture and microscopy	*Acanthamoeba* spp.	[134]
	Hospital Kuala Lumpur	10	1 (10%)									
	Hospital Tun Hussain Onn	30	2 (6.66%)									
	Private hospital	1	0 (0%)									
Jan-December 2003	Hospital Universiti Kebangsaan Malaysia	3	0 (0%)	74	11 (14.87%)	No data	Male = 1, female = 10	Contact lens	Corneal scrapings	Culture and microscopy	Group II (polyphagids)	[135]
	Hospital Kuala Lumpur	32	7 (21.88%)									
	Hospital Tun Hussain Onn	38	4 (10.53%)									
	Private hospital	1	0 (0%)									
2010-2012	Hospital Universiti Kebangsaan Malaysia			64	10 (15.6%)	No data	Male = 2, female = 8	Contact lens	Corneal scrapings	Culture and microscopy	*Acanthamoeba* spp.	[136]
2013-2015	Hospital Universiti Kebangsaan Malaysia	12	0 (0%)	62	14 (22.6%)	19-35	Male = 5, female = 9	Contact lens	Corneal scrapings	Culture and microscopy	*Acanthamoeba* spp.	[137]
	Hospital Sungai Buloh	8	0 (0%)									
	Private hospitals across Malaysia	42	14 (33.3%)									

In a 2013 study conducted at an optometry clinic and among students at Universiti Kebangsaan Malaysia (UKM), 10.6% (n = 7) out of 66 contact lens samples tested positive for Acanthamoeba (Table 3) [138]. Additionally, 13.5% (n = 7) of 25 contact lens users’ storage cases were contaminated with *Acanthamoeba* [138]. However, none of the 57 contact lens solution samples showed any presence of *Acanthamoeba* [138]. A more recent study conducted in 2020 at Universiti Teknologi MARA (UiTM) Puncak Alam further explored the prevalence of *Acanthamoeba* in contact lens paraphernalia. This study analyzed 180 samples each from contact lenses, storage cases, and solutions. The results revealed that 1.7% (n = 3) out of 180 contact lens samples were positive for *Acanthamoeba*, while 3.9% (n = 7) storage cases and 2.2% (n = 4) lens solution samples also tested positive [21]. Molecular analysis confirmed that all isolated strains belonged to the T4 genotype, commonly associated with AK [21]. The detection of *Acanthamoeba* in contact lenses and storage cases underscores the urgent need for proper hygiene practices among users, including meticulous cleaning and appropriate storage of lenses and cases. Furthermore, identifying the T4 genotype [21], known for its pathogenicity, highlights the severe risk of eye infections if proper precautions are not followed.

**Table 3:** Detection of Acanthamoeba spp. in contact lens equipment Notes: This table explains the prevalence of *Acanthamoeba* detected in contact lens equipment.

Year	Locations	Source	Total samples	Positive (%)	Sampling technique	Laboratory investigations	Acanthamoeba group/strains	References
2013	Optometry clinic and students of Universiti Kebangsaan Malaysia	Contact lens	66	7 (10.6%)	Swab	Culture and microscopy	*Acanthamoeba spp*.	[138]
		Contact lens storage cases	25	7 (13.5%)				
		Contact lens solutions	57	0 (0%)				
2020	Universiti Teknologi MARA Puncak Alam	Contact lens	180	3 (1.7%)	Swab	Culture, microscopy, and molecular	Group II = 11 and group III = 3 T4 in all isolated strains	[21]
		Contact lens storage cases	180	7 (3.9%)				
		Contact lens solutions	180	4 (2.2%)				

The link between AK and poor hygiene practices related to contact lens use is well-established. These practices are considered the primary risk factor for AK. Generally, some corneal trauma is necessary for *Acanthamoeba *trophozoites to establish an infection [29,139]. However, the tear film between the contact lens and the cornea may create a suitable environment for *Acanthamoeba *spp. to infect the eye. Symptoms of AK typically develop over several days to weeks and include intense eye pain, inflammation, photophobia, and stromal infiltration that can threaten vision. This condition is often misdiagnosed as a viral or bacterial infection, leading to delays in appropriate treatment [17,29,48,75].

Clinical cases of AK in Malaysia typically present with severe symptoms that require prompt and aggressive treatment (Table 4). Diagnosis is often confirmed through corneal scrapings and microscopy, with the presence of *Acanthamoeba *cysts and trophozoites [134-137]. Treatment regimens generally include a combination of topical medications, such as propamidine isethionate, chlorhexidine, and gentamycin, which target the amoeba's trophozoite and cyst forms [133,139]. However, due to the resilience of *Acanthamoeba* cysts, treatment can be prolonged and challenging, often requiring close monitoring and adjustments based on the patient’s response [17,19].

**Table 4:** Examples of Acanthamoeba infections documented in Malaysia

Location	Age	Sex	Source of infections	Clinical samples	Laboratory investigations	Diagnosis	Treatment	Outcome	References
Kuala Lumpur	40	Female	Contact lens	Corneal scraping	Culture and microscopy	*Acanthamoeba keratitis* with *Pseudomonas aeruginosa* and *E. coli*	Before diagnosis: Zovirax®; after diagnosis: gentamycin and homatropine eye drops, Neosporin, miconazole eye drops, and Brolene® (0.1% Propamidine isethionate)	No information	[133]
Kuala Lumpur	28	Male (Indonesian construction worker)	Rinsed his eyes with water from an open tank after exposure to sand and dust	Corneal scrapings	Culture and microscopy	Acanthamoeba keratitis	Topical propamidine isethionate, 0.02% chlorhexidine, and gentamycin	The condition improved, with the ulcer healing and vision getting better after 12 days. By day 32, the ulcer was nearly gone. Unfortunately, the patient left the hospital and didn’t return for follow-up.	[139]

On a global scale, *Acanthamoeba* infections, though relatively uncommon, are increasingly recognized as significant health risks, particularly concerning AK. The worldwide occurrence of AK is estimated at 2.9 cases per million individuals annually, with India reporting the highest rate at 15.2 cases per million per year [75]. Other countries with significantly high rates include Egypt, Portugal, and the UK, with 5.2, 5.0, and 4.3 cases per million annually, respectively [75].

Risk factors for AK vary considerably between countries. In developed nations, the leading risk factor, responsible for approximately 86% of cases, is using contact lenses for vision correction or cosmetic purposes [75]. On the other hand, developing countries such as India and China experience distinct primary risk factors. An Indian retrospective study conducted from 1999 to 2002 revealed that all patients diagnosed with AK were individuals working in agriculture who did not use contact lenses and had all suffered from eye injuries [140]. Another study conducted in China between 1997 and 2003 found that farmers accounted for 50.8% of AK cases, with students coming in second at 23.8% [18]. This finding highlights the vulnerability of different demographic groups to the infection. Compared to these countries, the prevalence of AK in Malaysia seems to be in the upper range, indicating a notable public health issue [134-137].

Although AK is the most reported *Acanthamoeba* infection, GAE is a rare yet highly fatal disease that predominantly affects immunocompromised individuals. The infection usually starts when *Acanthamoeba* cysts or trophozoites enter the body via inhalation or skin lesions, after which the amoebae travel to the brain [3,35,74]. This triggers a chronic inflammatory response, forming granulomas-necrotic tissue areas surrounded by immune cells [3,36,74,96,141]. GAE has an exceptionally high mortality rate of 97-98% despite its low global incidence [33]. The infection is frequently underdiagnosed and has been reported in individuals with conditions such as HIV/AIDS, organ transplant recipients, and cancer patients undergoing treatment [33,35,36,42,45,74]. While GAE primarily targets immunocompromised patients, it has also been documented in immunocompetent individuals. In the United States, 18 cases were reported between 1998 and 2006, with most being fatal. Austria and Germany each reported three cases, all with severe outcomes, including fatalities [3,142]. India documented 14 cases, reflecting a mix of survival and deaths in both immunocompetent and immunocompromised individuals [3]. China reported three post-mortem cases, all of which were fatal [86], while Thailand reported two fatal cases involving patients with severe underlying conditions [143]. Given the environmental prevalence of *Acanthamoeba* in Malaysia, the risk of GAE cannot be overlooked, especially with the high contamination rates in hospital water systems that could expose vulnerable patients.

Infections in other body parts, such as the skin and respiratory tract, are particularly concerning for immunocompromised populations. A study reported that from 1956 to 2020, the United States recorded 173 cases of non-keratitis *Acanthamoeba* infections [96]. GAE was the predominant manifestation, representing 71% of these cases. Other organs affected included the skin (CA) in 46% of cases, the sinuses in 29%, the lungs (AP) in 13%, and the bones (notably the hard palate) in 6% [96]. Nearly half of the patients experienced multiorgan involvement, often leading to fatal outcomes. The main risk factors for non-keratitis *Acanthamoeba* infections included immunocompromising conditions such as HIV infection (39%), cancer (28%), and solid organ or hematopoietic stem cell transplants (28%). Patients reported environmental exposures to soil (11%) and water (10%), with nasal irrigation (5%) linked explicitly to rhinosinusitis and GAE [96]. The extensive occurrence of *Acanthamoeba* in numerous environmental sources, such as soil, air conditioning systems, and water supplies, underscores the potential risk of infection despite no documented cases in Malaysia. The link between *Acanthamoeba* infections and environmental exposure, especially in areas like Malaysia with high humidity and water contamination, emphasizes the critical need for public health initiatives to enhance contact lens hygiene and minimize exposure to contaminated water. Countries with similar environmental conditions, such as Thailand and the Philippines, face comparable challenges in managing *Acanthamoeba* infections due to the difficulty in controlling environmental spread [45,144]. The persistent presence of *Acanthamoeba* in water systems, combined with the resilience of its cyst form, underscores the complexity of managing these infections globally, particularly in tropical regions like Malaysia, where the pathogen thrives in both natural and man-made environments.

Laboratory Approaches to Acanthamoeba Detection in Malaysia

**Figure 5:**
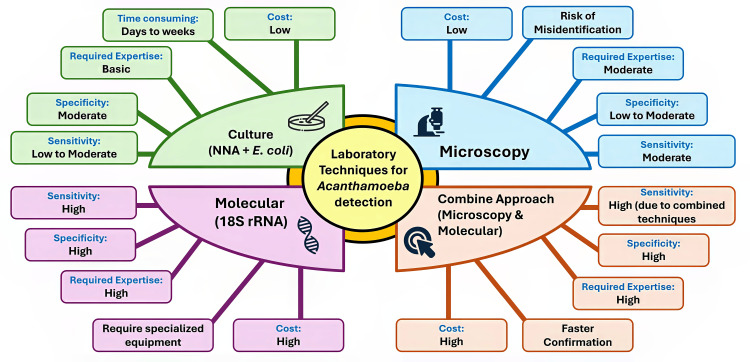
Detection techniques for Acanthamoeba Image Credit: Mohammad Wisman Abdul Hamid

Extensive research has focused on detecting and confirming (Figure 5) *Acanthamoeba* in environmental and clinical samples in Malaysia, utilizing a range of laboratory methods to ensure precise identification. The primary methods used include culture techniques, microscopy, and molecular approaches, all of which play crucial roles in clinical diagnostics and environmental surveillance.

Culture methods remain a foundational approach in *Acanthamoeba* detection. Samples from the environment, such as water, soil, and air conditioning dust, and clinical specimens, like corneal scrapings, are cultured on NNA plates inoculated with *E. coli*. This process encourages the growth of *Acanthamoeba* trophozoites and cysts, which can be identified microscopically. The culture method has been effectively used across various studies in Malaysia to identify *Acanthamoeba *spp. from a range of sources. For instance, research found that household water supply samples in Kuala Lumpur had a positivity rate of 2.4% (n = 1) out of 42 for *Acanthamoeba* spp. [4]. In comparison, another study found a higher positivity rate of 46.19% (n = 388) out of 840 swimming pool water samples from the same area [131].

Microscopy, particularly phase-contrast microscopy, is employed to observe *Acanthamoeba*’s characteristic double-walled cysts and motile trophozoites. This method is often used with culture techniques to verify if *Acanthamoeba* spp. is present. The efficacy of this combined approach is evident in various studies conducted across Malaysia. For example, a study using culture and microscopy achieved a detection rate of 100% (n = 20) for *Acanthamoeba* spp. in swabs collected from outdoor and indoor wall surfaces at the Medical Faculty of UM [5]. Similarly, in clinical settings, microscopy has been crucial in diagnosing AK, particularly in contact lens users, where the organism was detected in corneal scrapings with notable success rates across multiple studies.

The role of molecular techniques, particularly PCR, has become increasingly prominent in detecting and identifying *Acanthamoeba* at the strain level. Molecular methods offer higher specificity and sensitivity than traditional culture and microscopy techniques. For instance, a PCR-based investigation detected *Acanthamoeba* spp. in 24.9% (n = 45) out of 181 tap water samples and 70.2% (n = 40) out of 57 water samples from recreational sites throughout Peninsular Malaysia [127]. These findings highlight the ubiquity of *Acanthamoeba* in environmental water sources, which poses potential risks to public health, particularly for individuals who use contact lenses or engage in activities involving water exposure.

PCR-based genotyping has also been instrumental in identifying specific *Acanthamoeba* strains associated with different environments. Research identified several *Acanthamoeba *genotypes in Malaysia, with the T4 genotype being the most commonly isolated, especially in clinical keratitis cases [21]. A molecular technique study identified multiple genotypes, such as T3, T4, T5, T11, and T15, in water samples collected from hot springs in several states, including Perak, Selangor, and Pahang [128]. This genotypic diversity highlights the complexity of *Acanthamoeba *ecology and the need for continued molecular surveillance to understand different strains’ distribution and potential pathogenicity.

In clinical laboratories, the combination of culture, microscopy, and molecular methods has been proven effective in diagnosing *Acanthamoeba* infections. For example, a study conducted from 2013 to 2015 reported a positivity rate of 22.6% (n = 14) out of 62 in corneal scrapings from patients across several hospitals in Malaysia, with the identification of *Acanthamoeba* spp. confirmed through culture and microscopy [137]. The integration of PCR in these diagnostic workflows further enhances detection accuracy, allowing for the precise identification of *Acanthamoeba* strains, which is critical for guiding appropriate treatment strategies.

Environmental studies have similarly benefited from these combined laboratory approaches. Using culture and molecular methods has revealed a widespread presence of *Acanthamoeba* across various environmental settings in Malaysia. For example, a study detected *Acanthamoeba* spp. in all water samples from recreational rivers and wet soil samples in Selangor and Kuala Lumpur [6]. This extensive contamination highlights the environmental resilience of *Acanthamoeba *and the potential risks associated with exposure, particularly in recreational and public water sources.

Moreover, studies conducted in hot springs and recreational Beaches have consistently identified *Acanthamoeba*, with detection rates as high as 100% (n = 10) samples in some locations. The application of molecular methods in these studies has been crucial in determining the specific genotypes that vary depending on the geographical area and environmental conditions. For example, a study reported the detection of T4, T5, T11, T17, and T18 genotypes in water samples from multiple beaches across Selangor, Negeri Sembilan, and Pahang [129]. These findings are essential for public health surveillance, as specific genotypes, primarily T4, are linked to an increased risk of keratitis in humans [75].

Management of Acanthamoeba Infections

The management of *Acanthamoeba* infections, especially AK, in Malaysia, has advanced over time, blending local approaches with international treatment guidelines. AK, a severe and potentially vision-threatening corneal infection caused by the widespread *Acanthamoeba* spp., necessitates a treatment plan that addresses the amoeba’s active trophozoite and the more resistant cyst stages.

In Malaysia, the primary treatment regimen for AK involves the use of 0.02% chlorhexidine (a biguanide) and propamidine isethionate (a diamidine) [133,139]. Chlorhexidine is particularly valued for its effectiveness in penetrating the rigid cyst walls of *Acanthamoeba*, making it a cornerstone of AK treatment in the country. Research has shown that 0.02% chlorhexidine displays strong cysticidal properties, essential for eliminating the infection. Propamidine isethionate is another critical component of the treatment protocol, known for its ability to disrupt the cell membranes of *Acanthamoeba* trophozoites and cysts [145].

The treatment strategy in Malaysia aligns with international practices, mainly using biguanides and diamidines as first-line therapies, similar to protocols followed in the United States and the United Kingdom. The combination of these two agents has been the standard treatment for AK for several decades [2,146]. Additionally, the Malaysian approach includes broad-spectrum antibiotics such as gentamicin and ciprofloxacin, primarily used to address bacterial co-infections frequently encountered in AK patients [133,139]. This strategy is supported by studies demonstrating the effectiveness of these antibiotics in reducing bacterial load, which can exacerbate the severity of AK [2,147].

In contrast to AK, which is relatively well-documented and treated in Malaysia, GAE, a rare and fatal infection caused by *Acanthamoeba*, has not been reported in the country. Case reports primarily inform the treatment of GAE in survivors. The CDC recommends a treatment regimen typically involving five or six antimicrobial agents, including pentamidine, sulfadiazine, flucytosine, fluconazole, and miltefosine [96]. Beyond the primary treatment regimen, survivors have frequently been administered various empiric therapies, including acyclovir, albendazole, amphotericin B, doxycycline, ethambutol, ketoconazole, isoniazid, minocycline, pyrazinamide, posaconazole, rifampin, trifluoperazine, thioridazine, trimethoprim-sulfamethoxazole, voriconazole, and/or different steroids [73]. Even with these treatments, the success rate remains limited, mainly because the disease is often diagnosed in its later stages [3,73,86].

The challenge in treating both AK and GAE lies in the ineffectiveness of certain drugs against *Acanthamoeba* cysts. A study conducted in Malaysia found that 0.02% chlorhexidine digluconate showed the most substantial cysticidal effect, with 0.1% propamidine following closely behind. In contrast, gentamicin and ciprofloxacin were significantly less effective against *Acanthamoeba* cysts [147,148]. This finding aligns with global research indicating that many anti-*Acanthamoeba* agents, while effective against other pathogens, fail to kill *Acanthamoeba* cysts, highlighting the need for alternative treatment strategies, particularly in managing infections like GAE, where the prognosis is poor [73,96].

Advancements in Anti-Acanthamoeba Treatment Through Natural Products and Nanotechnology in Malaysia

Plant-derived compounds have been widely researched in Malaysia for their potential anti-*Acanthamoeba *properties. Betulinic acid, a plant-derived compound from *Pericampylus glaucus* (Broad-leaved Moonseed; Malay: Akar Menkunyit), a common Malaysian plant, demonstrated moderate efficacy against *A. triangularis* trophozoites isolated from environmental water samples, with a cytotoxic concentration (CC_50_) to inhibitory concentration (IC_50_) ratio of 3.75 µg/mL. This moderate efficacy suggests that betulinic acid may help treat trophozoite stages but may require a combination with other agents for full effectiveness against cysts​ [149]. Another compound from the same plant, Periglaucine A, exhibited a much lower efficacy with a CC_50_/IC_50_ of 170 µg/mL against the same strain, indicating that different components of the same plant can vary significantly in their effectiveness [150].

Further research into betulinic acid, enhanced by nanoparticle technology, demonstrated improved efficacy. When betulinic acid was combined with poly(DL-lactide-co-glycolide) nanoparticles, the CC_50_/IC_50_ improved to 5 µg/mL, indicating that nanoparticle delivery systems can significantly enhance the bioavailability and effectiveness of anti-*Acanthamoeba* agents​ [149]. Similarly, Periglaucine efficacy was enhanced using the same nanoparticle technology, with its CC_50_/IC_50 _improving to 25 µg/mL, though this is still relatively low compared to betulinic acid​ [149].

Ethyl acetate and butanol fractions from *Lonicera japonica* (Japanese Honeysuckle) demonstrated significant effects against *A. triangularis* trophozoites, with a marked reduction in the cyst-to-trophozoite ratio when treated with chlorogenic acid, a key component of *L. japonica*. This finding is promising as it suggests these plant extracts could play a role in reducing infection severity, though the complete eradication of cysts remains a challenge [151].

Cinnamic acid, derived from *Cinnamomum cassia*, showed enhanced activity against *A. castellanii* (ATCC 50492) when delivered using gold nanoparticles. The nanoparticle delivery system significantly improved the effectiveness of cinnamic acid, though, as with other treatments, complete eradication of cysts was not achieved​ [150]. This suggests that while nanoparticle technology enhances the efficacy of certain compounds, the resilience of *Acanthamoeba* cysts remains a considerable challenge.

Flavonoids such as naringin and hesperidin, derived from Citrus species, have also been explored. At a 50 µg/mL concentration, naringin, stabilized by gold nanoparticles with Tragacanth tree gum, eliminated the viability of *A. castellanii *[152]. Hesperidin showed similar efficacy at the same concentration when stabilized by silver nanoparticles with Acacia tree gum. These results are promising for treating trophozoites, but as with other agents, the challenge of completely eradicating cysts persists [152].

The use of nanoparticles in combination with traditional drugs has also been explored to increase their efficacy against *Acanthamoeba*. Silver nanoparticles have been utilized to boost the effectiveness of medications like diazepam, phenobarbitone, and phenytoin in combating *Acanthamoeba*. These combinations improved efficacy against trophozoites, though the impact on cysts was still limited, indicating that while nanoparticles can boost drug performance, they are not a complete solution to the problem of cyst resilience [153]. Gold nanoparticles have similarly been used to enhance the efficacy of antifungal agents like nystatin, fluconazole, and amphotericin B, with some success in reducing cyst viability. Still, again, total eradication has not been achieved [154].

**Figure 6:**
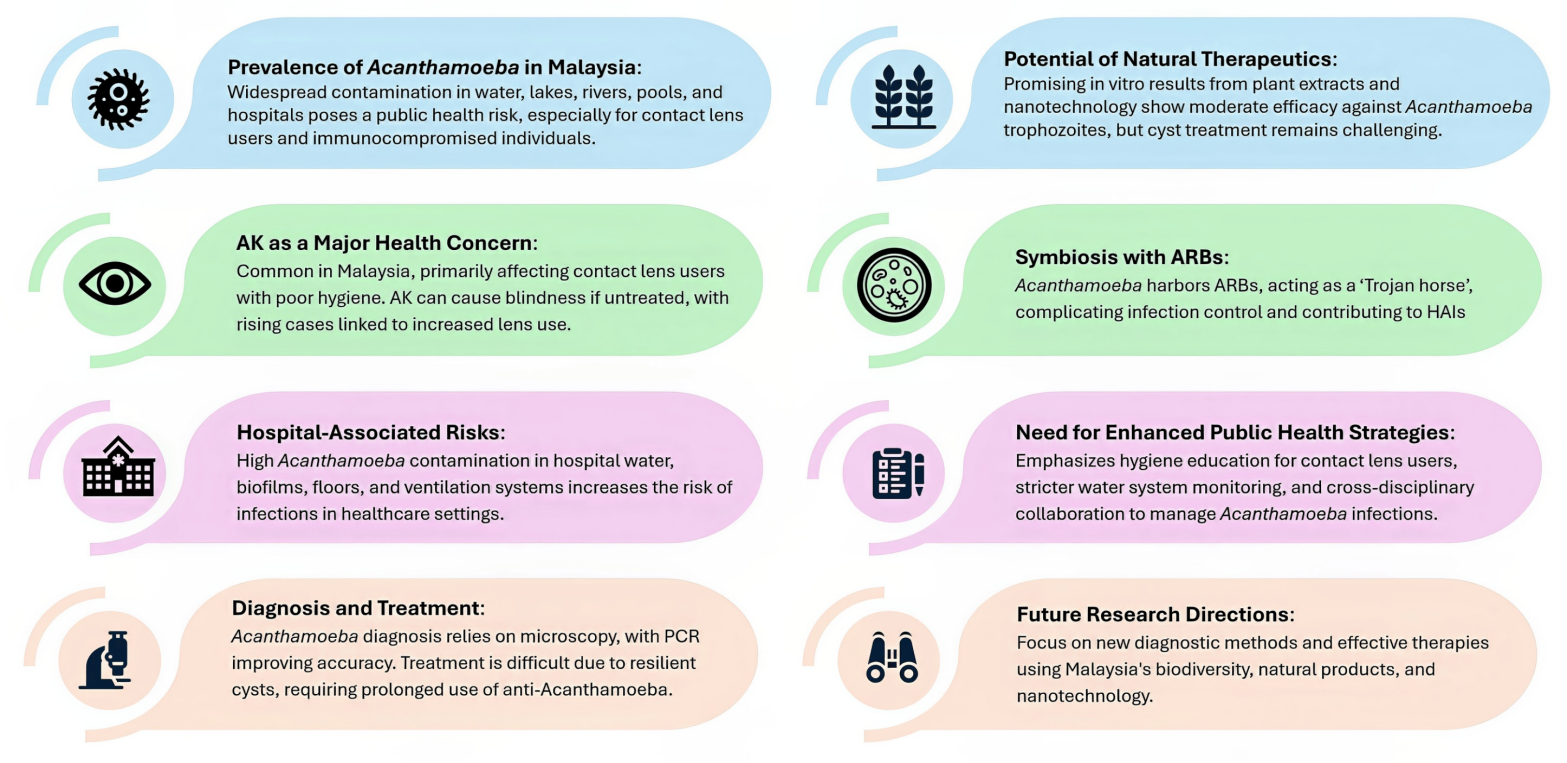
Key findings on the challenges in addressing Acanthamoeba issues in Malaysia Image Credit: Mohammad Wisman Abdul Hamid

Natural products from microorganisms and animals have also been explored. Supernatants from bacteria found in cockroach guts, including *Serratia marcescens *and *E. coli*, have demonstrated notable anti-*Acanthamoeba* activity against *A. castellanii *[155]. Crocodile (*Crocodylus palustris*) serum has effectively reduced trophozoite viability [156]. At the same time, sea sponge (*Aaptos aaptos*) extracts have shown potent activity with IC_50_ values between 0.615 and 0.876 µg/mL against clinical *A. castellanii *strains​ [157]. These findings suggest that natural products may complement more conventional treatments, though their effects on cysts require further investigation. The principal findings of this narrative review are depicted in Figure 6.

Future recommendations

**Figure 7:**
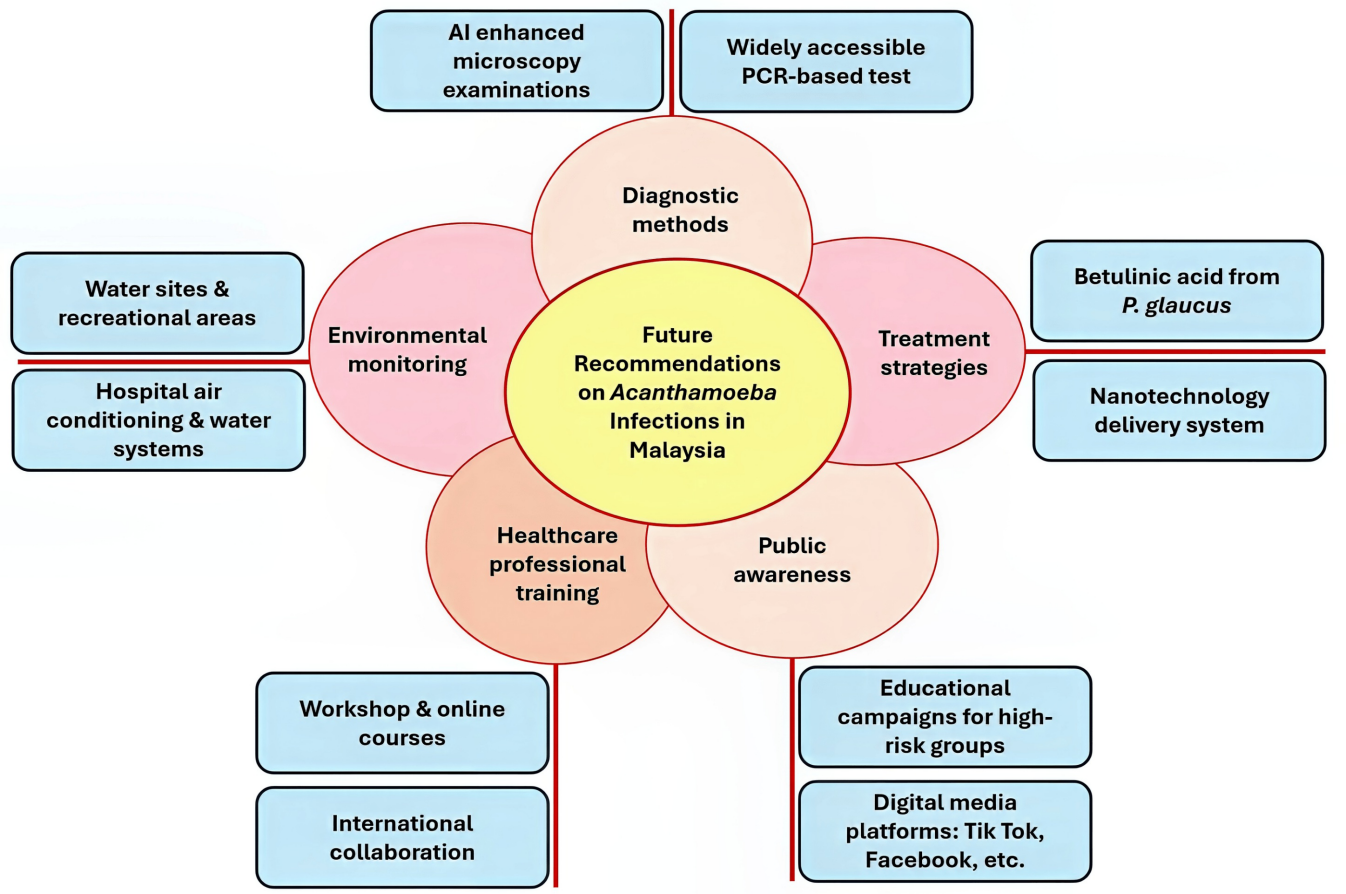
Recommendations for addressing Acanthamoeba infections in Malaysia Image Credit: Mohammad Wisman Abdul Hamid

Future research on *Acanthamoeba* infections in Malaysia should focus on advancing diagnostic methods, refining treatment strategies, and raising public awareness (Figure 7). A key priority is enhancing diagnostic accuracy through integrating artificial intelligence (AI) to analyze microscopy images, which can considerably improve the identification of *Acanthamoeba* species. Furthermore, ensuring the broader availability of advanced techniques such as PCR-based tests is crucial for the quicker and more accurate detection of *Acanthamoeba *in clinical and environmental samples.

Malaysia’s rich biodiversity also presents promising opportunities for natural therapeutics, with compounds like betulinic acid from *P. glaucus *showing potential in combating *Acanthamoeba* infections. Further research into these compounds and innovative delivery methods like nanotechnology could considerably enhance treatment efficacy.

Increasing healthcare professionals’ awareness of *Acanthamoeba *infections, especially its role as a “Trojan horse” for ARBs, is also crucial, as this complicates treatment. Continuous medical education and professional training programs should emphasize early detection and management, utilizing workshops, online courses, and collaboration with international experts to keep healthcare workers updated on the latest advancements. Public health initiatives should focus on high-risk groups, such as contact lens users, promoting proper hygiene practices through educational campaigns in schools, universities, clinics, and digital platforms like TikTok and Facebook.

Additionally, rigorous environmental monitoring and control in public spaces such as water recreational sites, beaches, and hot springs, as well as in hospitals, particularly centralized air conditioning and water systems, are crucial for preventing the spread of *Acanthamoeba* and ARBs. Interdisciplinary research and improved diagnostic and therapeutic strategies are essential for reducing the public health impact of *Acanthamoeba* infections in Malaysia. By aligning these efforts, Malaysia can more effectively combat these infections and enhance public health outcomes.

Limitations of the review paper

The studies primarily focus on urban areas like Kuala Lumpur, potentially overlooking rural regions where *Acanthamoeba* contamination may differ. The reliance on cross-sectional studies limits understanding of long-term infection trends. The review also emphasizes conventional diagnostic methods like microscopy, which are limited in sensitivity. While molecular techniques like PCR are discussed, challenges such as cost and accessibility are not fully explored. Additionally, the review lacks in vivo or clinical trial data to support the efficacy of potential treatments like plant-based therapies and nanotechnology. Public health awareness and infection control strategies are also not adequately addressed. Further research is necessary to gain a more comprehensive understanding of *Acanthamoeba* infections in Malaysia and to develop practical diagnostic and treatment approaches.

## Conclusions

*Acanthamoeba i*nfections in Malaysia present significant challenges that require a multifaceted approach involving advanced research, improved diagnostics, effective treatment strategies, and increased public and healthcare awareness. The organism's capacity to form resilient cysts and host ARBs makes diagnosing and treating these infections challenging, requiring continuous advancements in therapeutic and diagnostic methods. With the increasing incidence of AK, especially among contact lens users, it is crucial to develop more effective public health strategies to mitigate the risk of infection. Future research should explore novel therapeutic agents derived from Malaysia's biodiversity and utilize nanotechnology to enhance treatment efficacy. At the same time, efforts to make advanced diagnostic tools more accessible and to increase awareness among the public and healthcare professionals are critical for controlling the spread of *Acanthamoeba* infections. By addressing these challenges holistically, Malaysia can enhance its capacity to manage and prevent *Acanthamoeba* infections, thereby minimizing their impact on public health.
